# The Prevalence of Burnout and Mental Health Symptoms Among ICU Professionals in Greece: A National Comparative Study Between the COVID-19 Pandemic and Pre-pandemic Eras

**DOI:** 10.7759/cureus.84555

**Published:** 2025-05-21

**Authors:** Varvara Pakou, Ioannis Andrianopoulos, Mary Gouva, Elena Dragioti, Fotios Tatsis, Georgios Papathanakos, Asimenia Ntantana, Eleni Antoniadou, Fotini Fligou, Eirini Papageorgiou, Vasiliki Chantziara, Evaggelia Koutra, Ourania Mousafiri, Sofia Asoniti, Georgios Sidiras, Angeliki Giamarelou, Maria Nakou, Andreas Petsas, Athanasia Tsirogianni, Konstantinos Nianiopoulos, Eleni Papandreou, Aikaterini Panagiotakopoulou, Vasilios Koulouras

**Affiliations:** 1 Intensive Care Unit, University Hospital of Ioannina, Ioannina, GRC; 2 Research Laboratory Psychology of Patients, Families & Health Professionals, University of Ioannina, Ioannina, GRC; 3 Faculty of Medicine, School of Health Sciences, University of Ioannina, Ioannina, GRC; 4 Intensive Care Unit, Papageorgiou General Hospital of Thessaloniki, Thessaloniki, GRC; 5 Intensive Care Unit, Gennimatas General Hospital of Thessaloniki, Thessaloniki, GRC; 6 Department of Anesthesiology and Intensive Care Medicine, University Hospital of Patras, Rion, GRC; 7 Intensive Care Unit, Theagenio Cancer Hospital of Thessaloniki, Thessaloniki, GRC; 8 Intensive Care Unit, National and Kapodistrian University of Athens, Athens, GRC; 9 Intensive Care Unit, Naval and Veterans Hospital of Athens, Athens, GRC; 10 Intensive Care Unit, Chatzikosta General Hospital of Ioannina, Ioannina, GRC; 11 Intensive Care Unit, General Hospital of Corfu, Corfu, GRC; 12 Cardiac Care Unit, Evangelismos Hospital of Athens, Athens, GRC; 13 Intensive Care Unit, General Hospital of Kalamata, Kalamata, GRC; 14 Intensive Care Unit, University Hospital of Alexandroupolis, Alexandroupolis, GRC; 15 Intensive Care Unit, General Hospital of Patras Agios Andreas, Patras, GRC; 16 Intensive Care Unit, General Hospital of Lamia, Lamia, GRC; 17 Intensive Care Unit, University Hospital of Larissa, Larissa, GRC; 18 Intensive Care Unit, Agios Dimitrios General Hospital of Thessaloniki, Thessaloniki, GRC; 19 Intensive Care Unit, General Hospital of Eleusis Thriasion, Eleusis, GRC

**Keywords:** burnout, covid-19, intensive care, mental health, spirituality

## Abstract

Objective

This study aimed to assess the impact of the coronavirus disease 2019 (COVID-19) pandemic on burnout, mental health, and spirituality/religiosity among ICU professionals.

Methods

A multicenter, national, cross-sectional study was conducted in 31 adult ICUs across Greece. A compilation of three questionnaires assessing responders’ level of burnout [Maslach Burnout Inventory (MBI)], responders’ spirituality and religious attitudes [Spiritual and Religious Attitudes Questionnaire (SpREUK)], and finally, responders’ mental health [Symptom Checklist 90-R (SCL-90)] was distributed to ICU professionals in 2022. Responses were compared to those of a similar cohort of ICU professionals who had responded before the pandemic (2015).

Results

Overall, 1,013 ICU professionals participated (440 from the pre-pandemic period and 573 from the pandemic period). The prevalence of burnout increased during the pandemic (35.95 ±16.11%) compared to the pre-pandemic (30.62 ±15.39%, p<0.001) period. In particular, ICU professionals during the pandemic period scored worse across all three burnout categories: emotional exhaustion (25.80 ±10.93 vs. 22.27 ±10.96, p<0.001), depersonalization (10.40 ±6.51 vs. 8.68 ±6.25, p<0.001), and personal accomplishment (31.36 ±8.63 vs. 35.06 ±7.50, p<0.001) compared to the pre-pandemic period. In addition, during the pandemic period, participants reported experiencing more mental health symptoms and had a higher Global Severity Index (0.56 ±0.44 vs. 0.913 ±0.73, p<0.001), indicating a decline in mental health. Finally, participants during the pandemic era scored worse in the spirituality scale (overall score: 36.20 ±12.54 vs. 39.11 ±12.34, p<0.001), a significant mechanism associated with resilience and burnout risk factor.

Conclusions

The COVID-19 pandemic was associated with increased burnout, worsened mental health, and reduced spirituality among ICU professionals in Greece. These findings underscore the urgent need for targeted national and international policies and interventions to support the well-being and resilience of frontline healthcare workers.

## Introduction

Burnout is a syndrome characterized by work-related emotional exhaustion, depersonalization, and reduced personal accomplishment [1,2]. It is associated with low job satisfaction and increased turnover intention among healthcare professionals, as well as the reduction in patient satisfaction and quality of care [3]. Burnout appears to affect emergency department and intensive care professionals more commonly [3]. For intensive care professionals, in particular, burnout appears to be an ongoing epidemic as it is highly prevalent in both critical care nurses and physicians [4,5]. Up to 86% of intensive care nurses have experienced at least one symptom of burnout, while up to 45% of intensive care physicians experience symptoms of severe burnout [6,7]. Several risk factors for burnout have been identified, including individual characteristics and personality, working relationships, workload, working with dying patients, and end-of-life issues [6,8-11].

The outbreak of the coronavirus disease 2019 (COVID-19) pandemic due to the severe acute respiratory syndrome coronavirus 2 (SARS‑CoV‑2) and the subsequent overcrowding of ICUs has led to excessive workload and psychological stress for intensive care professionals [12,13]. Early reports suggest a high level of burnout, particularly among intensive care professionals attending to COVID-19 patients and being at high risk for burnout [14-20]. Nevertheless, apart from a single-center study [14], there is hardly any research comparing the prevalence and characteristics of burnout before and during the pandemic.

In addition, apart from burnout, the COVID-19 pandemic appears to have negatively affected intensive care professionals’ mental health, as early studies report a high prevalence of depression and post-traumatic stress disorder (PTSD) [20-22]. Nevertheless, evidence is lacking as to whether the mental health of intensive care professionals has worsened over the pandemic period. A potential increase in the prevalence of mental health disorders and burnout among intensive care professionals due to the COVID-19 pandemic may have led to an increase in patient safety incidents, functional impairment of intensive care professionals, and an increase in cost of care [3,5,21] and, it is critical implement strategies at national and international levels to address this issue.

In light of this, we endeavored to investigate the difference in prevalence and characteristics of burnout before and during the COVID-19 pandemic among intensive care professionals. The secondary objective of our study was to investigate possible differences in the mental health state and spiritual attitudes of intensive care professionals before and during the COVID-19 pandemic.

## Materials and methods

Study design and patient population

We conducted a national multicenter cross-sectional study from December 2021 to June 2022. A survey questionnaire was sent out to all ICUs in Greece. This questionnaire was addressed to all doctors (either in training or attending physicians), nurses, and nurse assistants working in these ICUs during that period.

The survey questionnaire wcomprised questions on demographics and socioeconomic status of the responder as well a compilation of three questionnaires assessing responders’ level of burn-out, using The Maslach Burnout Inventory (MBI), responders’ spirituality and religious attitudes, by using the Spiritual and Religious Attitudes Questionnaire (SpREUK) SF-10, developed by Arndt Büssing and colleagues, and finally, responders’ level of psychopathology by using the Symptom Checklist 90-R (SCL-90).

MBI [1] is a 22-item self-reported questionnaire assessing responders' level of burnout and incorporates three different aspects of burnout: emotional exhaustion, depersonalization, and personal exhaustion. We defined burnout as having an overall MBI cutoff of at least 34 for emotional exhaustion combined with a cutoff of at least 13 for depersonalization, according to the Greek evaluation of the scale [23]. SpREUK is a 15-item questionnaire exploring the spiritual and religious attitudes of respondents, suitable for both secular and religious societies [24]. The SCL-90-R is a 90-item questionnaire that records the existing psychopathology of the respondent by assessing nine different subtypes: somatization, obsessive-compulsive behaviors, depression, aggression, anxiety, interpersonal sensitivity, paranoid ideation and psychotism, and other disorders (i.e., sleeping or eating disorders) [25].

To compare the prevalence and characteristics of burnout, mental health, and spirituality among intensive care professionals during and before the pandemic period, we retrospectively used data from a previous similar national cross-sectional study that we conducted from June to December 2015. During that period, intensive care professionals answered a similar set of questionnaires that included the MBI, the SpREUK, and the SCL-90-R questionnaire.

Statistical analysis

Descriptive statistics were used to report the results of the study population. Kolmogorov-Smirnov test and Shapiro-Wilk test were used to assess the normality of distribution. Comparisons between groups were performed using the chi-square test for categorical variables, the Mann-Whitney U test for non-parametrical continuous variables with two degrees of freedom, and the Kruskal-Wallis test for higher degrees of freedom. Correlation analysis of quantitative variables was performed using Pearson’s r correlation coefficient. The credibility of psychometric tests was assessed by Cronbach's α value. Statistical significance was set at a p-value less than 0.05. SPSS Statistics (IBM Corp., Armonk, NY) program was used for all analyses.

Ethical approval

The questionnaires were filled out anonymously, and data were collected anonymously. The institutional review board approved data collection (approval no 1050/2021) and waived the need for informed consent.

## Results

Overall, 1,013 ICU professionals participated in the study: 440 in the pre-pandemic period and 573 in the pandemic period surveys. The response rate was 61.6% (440/714) and 47.9% (573/1196) for the pre-pandemic and the pandemic period, respectively. The mean age of the pre-pandemic and pandemic study groups was 40.82 ±7.19 and 41.60 ±8.33 years, respectively (p 0.122). There was no statistically significant difference in the two study groups in terms of age, gender, profession, and marital status (Table 1).

**Table 1:** Baseline characteristics of the participants SD: standard deviation; χ^2^p: Pearson's chi-squared test

Parameter	Pre-pandemic survey (n=440)	Pandemic survey (n=573)	
Age, mean ±SD	40.82 ±7.19	41.60 ±8.33	t-statistic=1.550, p=0.122
Female sex, n (%)	301 (72.0%)	388 (68.2%)	χ^2^p=1.668, p=0.197
Male sex, n (%)	117 (28.0%)	181 (31.8%)	
Profession, n (%)			
Physician	149 (34.0)	167 (29.5)	Fisher's exact test=5.532, p=0.137
Nurse	234 (53.4)	292 (51.6)	
Assistant nurse	46 (10.5)	84 (15.2)	
Other critical care clinicians	9 (2.1)	23 (4)	
Marital status, n (%)			
Unmarried	130 (29.9)	203 (36.1)	Fisher's Exact Test = 4.512, p =0.205

Overall, the prevalence of burnout increased from 30.62 ±15.39% during the pre-pandemic period to 35.95 ±16.11% during the pandemic period (p<0.001). As shown in Table 2, there was an increase in the burnout score in the ICU professionals during the pandemic period across all three burnout dimensions. In addition, an increase in the percentage of high-level burnout across all three dimensions of burnout was also noted in the pandemic period: emotional exhaustion (21.8% vs. 15%, p<0.001), depersonalization (36% vs. 26.8%), and personal accomplishment (73.5% vs. 59.1%, p<0.001) for the pandemic and the pre-pandemic periods, respectively (Table 2).

**Table 2:** Comparison of the prevalence of burnout levels between the pre-pandemic (2015) and pandemic (2022) surveys

Subscales of burnout	Level of burnout	Participants		
		2015, n (%)	2022, n (%)	
Emotional exhaustion	Low burnout (≤16)	163 (37.0)	116 (20.2)	Fisher's exact test=36.038, p<0.001
	Moderate burnout (17-33)	211 (48.0)	332 (57.9)	
	High burnout (≥34)	66 (15.0)	125 (21.8)	
Depersonalization	Low burnout (≤4)	145 (33.0)	120 (20.9)	Fisher's exact test=20.607, p<0.001
	Moderate burnout (5-12)	177 (40.2)	247 (43.1)	
	High burnout (≥13)	118 (26.8)	206 (36.0)	
Personal accomplishment	Low burnout (≤39)	158 (35.9)	134 (23.4)	Fisher's exact test=23.281, p<0.001
	Moderate burnout (30-38)	22 (5.0)	18 (3.1)	
	High burnout (≥29)	260 (59.1)	421 (73.5)	

Table 3 shows the results of the psychopathology and the religious and spiritual attitudes scales. ICU professionals in the pandemic period scored higher across all subscales of the psychopathology scale, indicating a worsening of ICU professionals’ mental health during the pandemic period. In addition, ICU professionals during the pandemic period scored lower in the religious and spiritual attitudes scale, suggesting a decline in ICU professionals’ spirituality (Table 3).

**Table 3:** Psychopathology, religious and spiritual attitudes and burnout questionnaire scores in the pre-pandemic (2015) and pandemic (2022) surveys SD: standard deviation

	Participant scores, mean ±SD		t-statistic	P-value
	2015	2022		
Psychopathology Scale - Symptom Checklist 90-R (SCL-90)				
Somatization	8.12 ±7.63	11.80 ±9.69	-6.594	<0.001
Obsessive-compulsive behaviors	7.42 ±5.88	11.23 ±7.98	-8.436	<0.001
Interpersonal sensitivity	5.55 ±4.85	8.68 ±7.00	-8.043	<0.001
Depression	8.06 ±7.52	12.68 ±10.38	-7.903	<0.001
Anxiety	4.29 ±5.28	8.16 ±8.03	-8.776	<0.001
Hostility	3.33 ±3.46	5.74 ±5.27	-8.328	<0.001
Phobic anxiety	1.26 ±2.58	3.98 ±5.46	-9.655	<0.001
Paranoid ideation	4.81 ±3.83	6.17 ±5.11	-4.696	<0.001
Psychoticism	3.25 ±3.29	6.52 ±7.51	-8.550	<0.001
Global Severity Index	0.56 ±0.44	0.913 ±0.73	-8.903	<0.001
Positive System Distress Index	1.51 ±0.39	1.65 ±0.52	-4.857	<0.001
Positive symptoms, total	31.17 ±18.53	45.92 ±25.66	-10.208	<0.001
Eysenck Personality Questionnaire (EPQ)				
Extraversion	14.09 ±3.89	12.01 ±4.28	7.972	<0.001
Neuroticism	10.01 ±4.63	10.89 ±4.57	-2.983	0.003
Psychotism	3.66 ±2.05	5.87 ±3.95	-10.614	<0.001
Lying	14.15 ±3.40	13.96 ±3.20	0.879	0.385
Spiritual and Religious Attitudes in Dealing with Illness Questionnaire (SpREUK-SF-15)				
Total score	39.11 ±12.34	36.20 ±12.54	3.630	<0.001
Search for support/access	11.94 ±4.99	10.97 ±4.73	3.105	0.002
Trust in higher guidance/source	12.92 ±4.69	12.14 ±4.64	2.625	0.009
Reflection	14.25 ±4.54	13.09 ±4.83	3.809	<0.001
Burnout Questionnaire (Maslach Burnout Inventory – MBI)				
Emotional exhaustion	22.24 ±10.96	25.80 ±10.93	-5.109	<0.001
Depersonalization	8.66 ±6.25	10.40 ±6.51	-4.255	<0.001
Personal accomplishment	35.08 ±7.50	31.36 ±8.63	7.154	<0.001
Overall burnout (emotional exhaustion + depersonalization)	30.62 ±15.39	35.95 ±16.11	-5.314	<0.001

Tables 4, 5 present findings from a comparative analysis of burnout among doctors and nurses across two time points: pre-pandemic (2015) and during the pandemic (2022). The data revealed a significant rise in emotional exhaustion for both groups, with higher levels recorded in 2022, indicating a pronounced increase in the demands on healthcare professionals. Depersonalization also showed a statistically significant rise from 2015 to 2022 for both doctors and nurses. This shift suggests that the pandemic milieu intensified emotional distancing among healthcare providers.

**Table 4:** Comparison of burnout questionnaire (Maslach Burnout Inventory – MBI) scores between the pre-pandemic (2015) and pandemic (2022) surveys SD: standard deviation

	Doctors				Nurses			
	2015, mean ±SD	2022, mean ±SD	t-statistic	P-value	2015, mean ±SD	2022, mean ±SD	t-statistic	P-value
Emotional exhaustion	18.51 ±10.61	24.78 ±10.69	-5.213	<0.001	24.41 ±10.59	26.41 ±11.25	-2.299	0.022
Depersonalization	8.59 ±5.77	10.24 ±6.89	-2.280	0.023	8.76 ±6.55	10.68 ±6.44	-3.741	<0.001
Personal accomplishment	35.95 ±7.67	33.42 ±7.10	3.034	0.003	34.45 ±7.37	30.15 ±9.14	6.427	<0.001
Overall burnout (emotional exhaustion + depersonalization)	26.92 ±15.18	34.81 ±16.26	-4.445	<0.001	32.81 ±15.15	36.80 ±16.34	-3.187	0.002

**Table 5:** Comparison of the prevalence of burnout levels between the pre-pandemic (2015) and pandemic (2022) surveys

Prevalence of burnout										
Subscales of burnout	Level of burnout	Participants			Doctors			Nurses		
		2015, n (%)	2022, n (%)		2015, n (%)	2022, n (%)		2015, n (%)	2022, n (%)	
Emotional exhaustion	Low burnout (≤16)	163 (37.0)	116(20.2)	Fisher's exact test=36.038, p<0.001	80 (53.7)	41 (24.6)	Fisher's exact test=30.015, p<0.001	78 (27.9)	73 (19.4)	Fisher's exact test=7.155, p<0.028
	Moderate burnout (17-33)	211 (48.0)	332 (57.9)		58 (38.9)	96 (57.5)		148 (52.9)	211 (56.1)	
	High burnout (≥34)	66 (15.0)	125 (21.8)		11 (7.4)	30 (18.0)		54 (19.3)	92 (24.5)	
Depersonalization	Low burnout (≤4)	145 (33.0)	120 (20.9)	Fisher's exact test=20.607, p<0.001	39 (26.2)	46 (27.5)	Fisher's exact test=9.004, p<0.011	101 (36.1)	68 (18.1)	Fisher's exact test=26.867, p<0.001
	Moderate burnout (5-12)	177 (40.2)	247 (43.1)		75 (50.3)	59 (35.3)		98 (35.0)	167 (44.4)	
	High burnout (≥13)	118 (26.8)	206 (36.0)		35 (23.5)	62 (37.1)		81 (28.9)	141 (37.5)	
Personal accomplishment	Low burnout (≤39)	158 (35.9)	134 (23.4)	Fisher's exact test=23.281, p<0.001	64 (43.0)	48 (28.7)	Fisher's exact test=7.072, p<0.027	87 (31.1)	75 (19.9)	Fisher's exact test=14.473, p<0.001
	Moderate burnout (30-38)	22 (5.0)	18 (3.1)		6 (4.0)	7 (4.2)		15 (5.4)	11 (2.9)	
	High burnout (≥29)	260 (59.1)	421 (73.5)		79 (53.0)	112 (67.1)		178 (63.6)	290 (77.1)	

In terms of personal accomplishment, both groups reported a marked decrease in 2022, reflecting a diminished sense of professional efficacy and success. The combined burnout score, which aggregates emotional exhaustion and depersonalization, highlights a notable increase in overall burnout levels in 2022 for both professional groups (Table 4). Analysis of burnout levels by category further underscores these trends. For emotional exhaustion, there was a substantial increase in the proportion of both doctors and nurses experiencing high burnout, with a corresponding decline in those with lower burnout levels. Depersonalization also rose significantly, with more healthcare professionals reporting moderate to high burnout, while the number experiencing low burnout levels declined. Regarding personal accomplishment, most doctors and nurses moved into the high-burnout category, with considerably fewer remaining in the low-burnout category (Table 5).

In 2015, doctors reported an average score of 22.24, whereas nurses reported 24.41; by 2022, these levels increased significantly for both groups, reaching 25.80 for doctors and 26.41 for nurses (p<0.001). Depersonalization scores were initially similar between the two groups in 2015 but showed a more pronounced rise among nurses by 2022. Nurses’ depersonalization scores shifted from 8.76 in 2015 to 10.68 in 2022, while doctors increased from 8.66 to 10.40 (p<0.001). Regarding personal accomplishment, both doctors and nurses experienced declines from 2015 to 2022, though the decrease was more substantial among nurses. Doctors’ scores fell from 35.08 to 31.36, whereas nurses’ scores dropped from 34.45 to 30.15. In terms of overall burnout, both groups experienced statistically significant increases. Doctors’ combined scores rose from 30.62 in 2015 to 35.95 in 2022, and nurses' from 32.81 to 36.80. When examining burnout categories, the shift to high-burnout levels was more prominent among nurses. A significant proportion of nurses moved from low to high categories in both emotional exhaustion and personal accomplishment, indicating a sharper increase in burnout levels compared to doctors (Tables 4, 5).

## Discussion

Our study involving 1,013 ICU professionals from 31 ICUs showed that during the COVID-19 pandemic, there was a significant increase in the prevalence of burnout compared to the pre-pandemic period. In addition, our study showed a decline in ICU professionals’ mental health and spirituality compared to the pre-pandemic survey. To the best of our knowledge, this is the first study performed at a national level that directly compares burnout prevalence among ICU professionals before and during the pandemic. Our study’s results align with preliminary reports [14-20]. Nevertheless, only one single-center study has so far compared the prevalence of burnout during the pandemic with that before the pandemic [14]. Moll et al. in their single-center study showed an increase in the prevalence of burnout among ICU professionals during the pandemic, with the nurses having the highest impact. While neither study addressed risk factors for the increase of burnout during the COVID-19 pandemic, other studies addressing this issue pointed to the increase in workload, the lack of personal protective equipment (PPE), and fear of acquiring the disease as risk factors for burnout [26,27].

Apart from an increase in the prevalence of burnout, our study showed a decline in mental health as indicated by the increase in most of the psychopathological subscales. To the best of our knowledge, this is the first study that compared the prevalence of psychopathology among ICU professionals before and during the pandemic. Preliminary reports have shown high prevalence of depression, anxiety, PTSD, and insomnia among healthcare professionals during the COVID-19 pandemic [28] and in ICU professionals in particular [20,21]. Our study is the first one to confirm an increase in the prevalence of most mental health symptoms among ICU professionals during the COVID-19 pandemic period. The increase in the prevalence of anxiety and depression is not surprising as they have similar risk factors with burnout, and there is also a strong correlation between depression and anxiety, with the latter [29,30]. Therefore, the decline in mental health may be partially responsible for the increase in burnout and vice versa.

Interestingly, spirituality also appeared to be affected by the COVID-19 pandemic. This phenomenon is relatively understudied, as most studies have focused on the role of spirituality in patient management during the COVID-19 pandemic. Our study showed that ICU professionals’ religiousness and spirituality appear to have declined during the pandemic. Taken together, the facts that spirituality is a mechanism of resilience and coping in stressful times [31], and that it is associated with burnout-as demonstrated by a previous study from our research group [24], suggest that the observed decline in spirituality during the pandemic may be linked to the increased prevalence of burnout among ICU professionals. However, the cross-sectional design does not allow for causal inferences. Also, our study did not control for potential confounding factors such as individual coping strategies, access to psychological or pastoral support, or specific institutional interventions implemented during the pandemic. These factors may have influenced both participants’ levels of spirituality and their experience of burnout. Future studies should aim to account for these variables to better understand the relationship between spirituality and professional well-being during times of crisis.

To summarize, our study showed a multi-level effect of the COVID-19 pandemic on the mental health of ICU professionals: an increase in the prevalence of burnout, accompanied by an increase in the prevalence of mental health symptoms and reduced spirituality, which has been a significant mechanism of resilience. The rise of this “hidden health crisis” has a significant impact on both the health of healthcare professionals as well as on patients’ outcomes and quality of care. New national and international strategies are urgently needed to confront this problem [32].

Limitations

Our study has several limitations. Firstly, it was conducted in Greece, and results may not be generalizable to other countries. However, as a multicenter study conducted at a national level within a European healthcare context, the findings may be relevant to countries with similar ICU structures and cultural settings. Second, although the overall response rate was acceptable, the use of self-reported questionnaires (MBI, SCL-90-R, and SpREUK) introduces potential information bias, including underreporting or overreporting of burnout and mental health symptoms. This is particularly relevant given that data collection took place during a high-stress period (i.e., the COVID-19 pandemic), increasing the risk of recall and social desirability bias. Additionally, the response rate was notably lower during the pandemic compared to the pre-pandemic survey (47.9% vs. 61.6%), which could have introduced selection bias. It is possible that ICU professionals more severely affected by burnout or psychological distress were either more inclined to participate or, conversely, too overwhelmed to respond, potentially impacting the representativeness of the sample.

Furthermore, as nearly six years had passed between the two survey periods, some participants may not have completed both questionnaires; however, no significant differences were observed in baseline characteristics between respondents. Of note, the study did not control for potential confounding variables such as individual coping mechanisms, access to mental health or spiritual support, or workplace interventions during the pandemic, which could have influenced both burnout and spirituality levels. We also did not collect data on workload metrics such as patient-to-staff ratios or overtime hours, which are key factors influencing burnout and may have changed substantially during the pandemic. Furthermore, while our findings suggest a decline in spirituality among ICU professionals, the study did not directly assess whether this decline was associated with burnout or mental health symptoms. Spirituality is a multifaceted and culturally sensitive construct, and although the SpREUK SF-10 is a validated instrument, it may not fully capture the breadth and depth of spiritual experience in this population. Finally, the cross-sectional design of the study does not allow for causal inferences, and further longitudinal research is needed to clarify potential relationships between spirituality, burnout, and mental health outcomes.

## Conclusions

The findings of our multicenter national study demonstrated an increase in burnout among ICU professionals during the COVID-19 pandemic. The study also showed an increase in the prevalence of reported mental health symptoms and a decrease in spirituality among ICU professionals. Implementation of new national and international policies is required to address this ongoing healthcare crisis.
